# Atrial Myxoma Presenting With Hemorrhage and Multifocal Infarcts in the Brain of a 59-Year-Old Man: An Uncommon Outcome for the Most Common Primary Heart Neoplasm

**DOI:** 10.7759/cureus.41323

**Published:** 2023-07-03

**Authors:** Arthur M Samia, Philip J Boyer

**Affiliations:** 1 Dermatology, University of Florida, Gainesville, USA; 2 Pathology and Laboratory Medicine, Vidant Medical Center/East Carolina University, Greenville, USA

**Keywords:** atrial myxoma, cardiac tumors, metastasis, brain tumors, neoplasia, ischemic stroke, hemorrhagic stroke, embolic infarction

## Abstract

Atrial myxomas are the most common primary neoplasm of the heart. Due to their mass effect, they may lead to dysfunction of the heart or mitral valve. Rarely, neoplastic fragments may embolize or a thrombus secondary to stasis may form, which can infarct downstream structures (e.g., the brain). We report the case of a 59-year-old man presenting with headaches, visual changes, and word-finding difficulty secondary to multifocal brain lesions that were identified on computed tomography and magnetic resonance imaging. After an extensive workup, the etiology of the patient’s neurological symptoms was determined to be embolization from a large atrial myxoma (2.3x3.5 cm). Histologic and immunohistochemical examination of the atrial myxoma and largest brain lesion yielded similarities, including the presence of spindle-shaped and stellate cells, myxoid regions, Alcian blue pH 2.5 positivity, calretinin positivity, cluster of differentiation 34 (CD34) positivity, and cluster of differentiation 68 (CD68) negativity. This case was remarkable due to the patient’s late presentation, the large size of the atrial myxoma, the presence of abundant cerebral hemisphere and cerebellar lesions, and the histologic comparison of the heart and brain lesions. Atrial myxomas have been reported from childhood to late adulthood and when symptoms typically present clinically due to the mass effect. However, neurologic manifestations from embolization or thrombus formation can occur, as in the present case. Therefore, considering the presence of atrial myxomas is important in patients with neurologic manifestations and heart murmurs.

## Introduction

Brain infarcts are typically either ischemic or hemorrhagic. Ischemic infarcts can be focal or global, appear pale, and may have hemorrhagic conversion [1]. Non-neoplastic causes of multiple hemorrhagic or ischemic brain infarcts may be traumatic or vascular in origin [1]. Some vascular causes include thromboembolism, atheroembolism, or vegetation embolism [1]. Neoplastic causes are most commonly metastatic, compared to primary or hematopoietic neoplasia [2]. Histopathologic evaluations of metastatic disease could show epithelial/epithelioid cells (e.g., carcinoma), spindle cells (e.g., melanoma or sarcoma), or small round blue cells (e.g., small cell carcinoma, melanoma, sarcoma, or germ cell tumors) [3]. Overall, the lungs are the most common source of metastasis to the brain [2]. The true prevalence of brain metastasis is not well known; however, it has been estimated to be around 9.6%, and metastasis is significantly more common than any other primary brain cancer [4]. The common origins of brain metastases, in order of most to least common, are lung cancer (19.9%), melanoma (6.9%), renal cancer (6.5%), breast cancer (5.1%), and colorectal cancer (1.8%) [4]. The incidence of brain metastases ranges from approximately 8.3 to 14.3 per 100,000 people; however, this number is likely an undershoot due to the increased lifespan of patients with cancer that results from earlier detection and better systemic therapies [2].

Cardioembolic strokes most commonly result from thromboembolism, atheroembolism, or vegetation embolism [1]. The most common cause of cardioembolic stroke worldwide is thromboembolism secondary to atrial fibrillation [1]. Other most common risk factors for cardioembolic strokes include systolic heart failure, recent myocardial infarction, patent foramen ovale, aortic arch atheroma, prosthetic heart valves, and infective endocarditis [1]. Other less common risk factors for cardioembolic strokes include papillary fibroelastomas, atrial myxomas, and mitral calcification, each of which accounts for fewer than 1% of cardioembolic strokes [5]. Here, we report the case of a man with symptoms of multifocal brain lesions and subsequent identification of a large atrial myxoma.

## Case presentation

**Figure 1:**
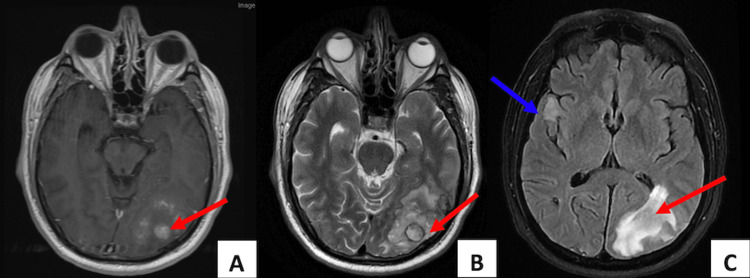
T1 (A), T2 (B), and flair (C) MRIs of the brain reveal a circumscribed mass (red arrows) in the left occipital lobe that is contrast-enhanced and with surrounding edema. Multiple ischemic or hemorrhagic lesions were noted in the cerebral hemispheres and cerebellum (blue arrow). MRI: magnetic resonance imaging

A 59-year-old man presented to the clinic with complaints of persistent headaches, visual changes, fatigue, and word-finding difficulty. He had no significant past medical history and did not take any medications. A cardiac examination revealed an early diastolic low-pitched murmur just after the second heart sound (S2). Neurologic examination showed 3+ reflexes on the left upper and lower extremities and gait instability. His physical and neurologic examinations were otherwise unremarkable, and his vital signs were within normal limits. Further workup included imaging of the head using computed tomography (CT) and magnetic resonance imaging (MRI), which identified a left occipital lobe mass measuring 2.0x2.1x2.3 cm with surrounding subacute and chronic hemorrhage, multiple other hemorrhagic lesions in the cerebral hemispheres and cerebellum, and multiple chronic lacunar infarcts (Figure 1 A-C).

**Figure 2:**
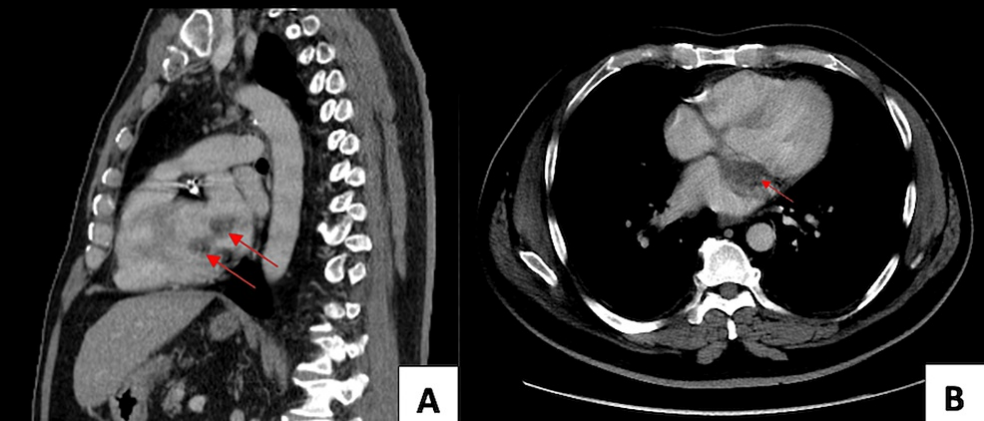
Transverse (A) and axial (B) CT scans identifying a low-attenuating lobular region in the left atrium and left ventricle, centered at the left mitral valve, and measuring 2.3x3.5 cm (red arrows). CT: computed tomography

**Figure 3:**
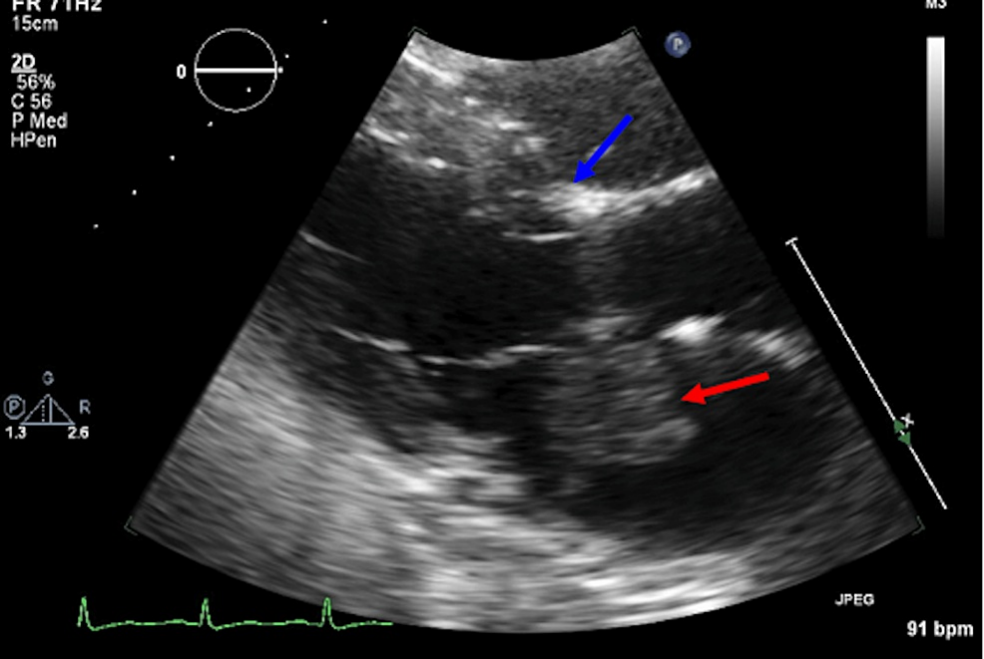
Echo identifies a large mass attached to the atrial septum (blue arrows), measuring 3.4x3.4 cm, very mobile, and entering the left ventricle with each diastolic cycle (red arrow). Echo: echocardiography

Given concerns for a metastatic disease, CT scans of the chest, abdomen, and pelvis were undertaken, which were initially read as negative for a primary or metastatic disease. A secondary review was initiated by the clinical neurosurgical service, which identified a lobular, low-attenuation left atrial and left ventricular lesion centered near the left mitral valve (Figure 2 A-B). Echocardiography (echo) confirmed this finding by identifying a lesion attached to the atrial septum, which was freely mobile, and entered the left ventricle with each diastolic cycle (Figure 3).

**Figure 4:**
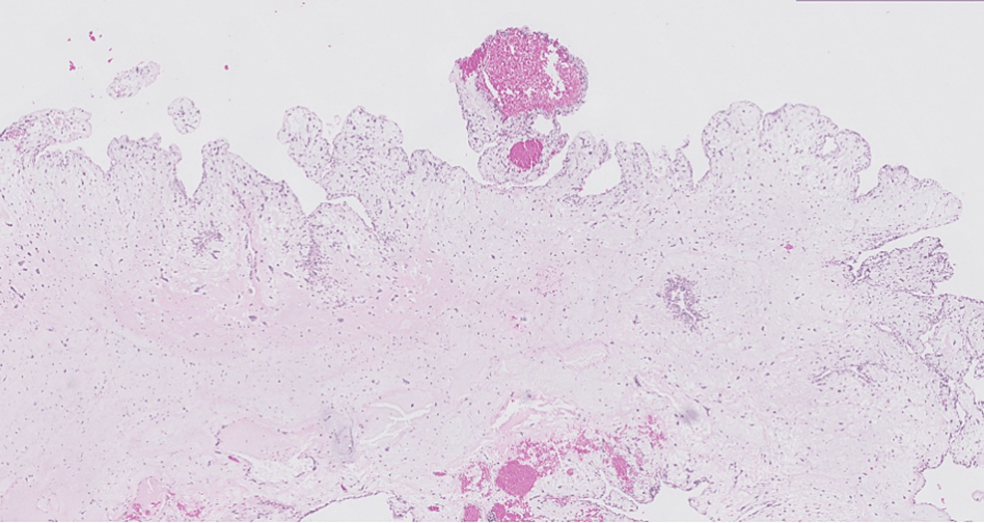
A low-magnification view of the left atrial-ventricular mass shows low cellularity and abundant extracellular matrix.

**Figure 5:**
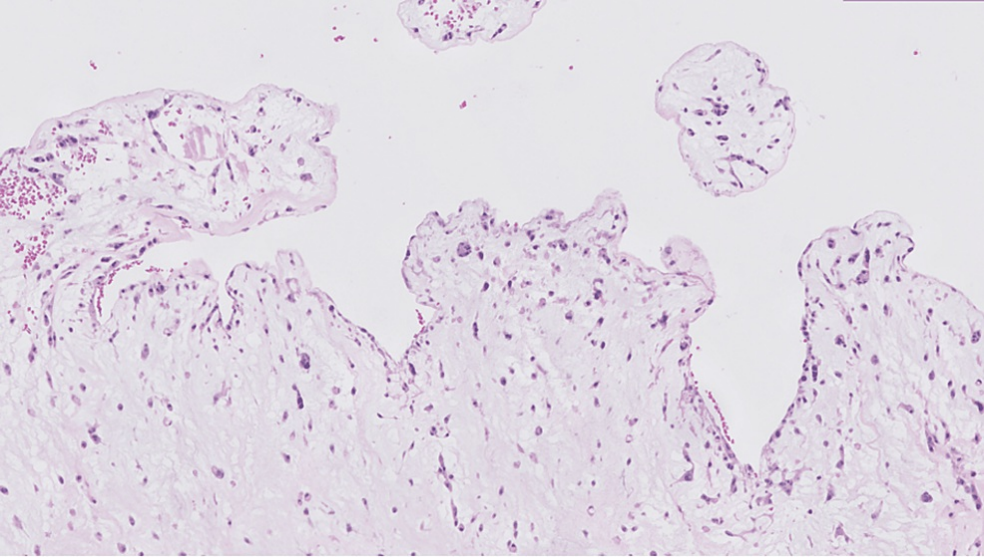
A medium-magnification view of the left atrial-ventricular mass shows papillary projections from the surface.

**Figure 6:**
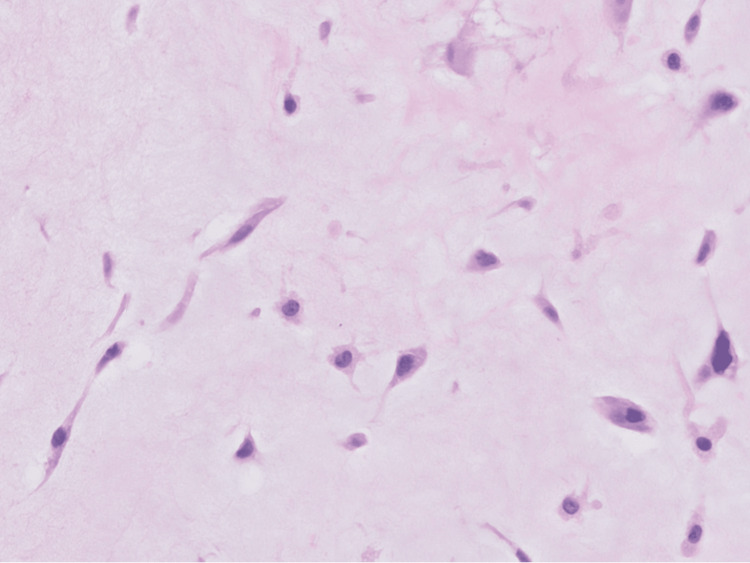
A high-magnification view of the left atrial-ventricular mass shows spindle-shaped cells, stellate cells, myxoid stroma, and no mitotic figures.

**Figure 7:**
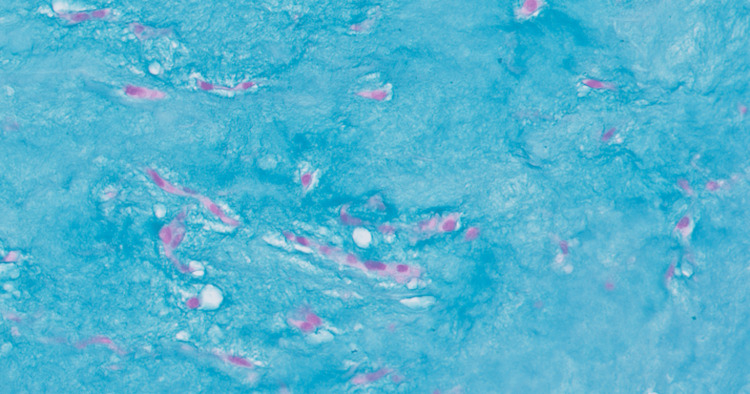
Alcian blue pH 2.5 stain of the left atrial-ventricular mass. The extracellular matrix contains abundant acid mucin and mucopolysaccharides.

**Figure 8:**
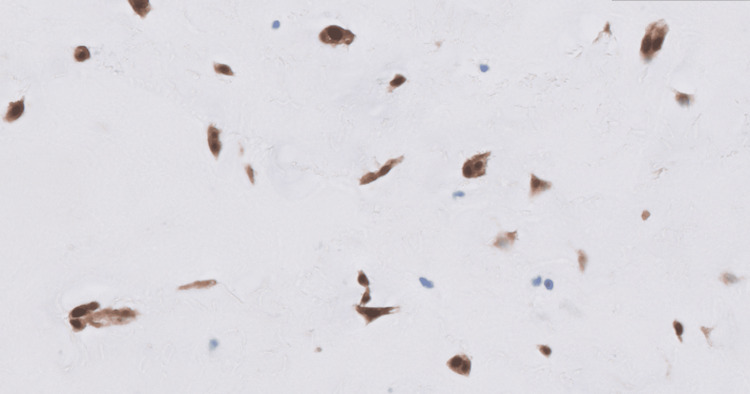
Calretinin stain of the left atrial-ventricular mass. Immunohistochemistry is positive for calretinin and CD34 and negative for CD68. CD34: cluster of differentiation 34; CD68: cluster of differentiation 68

An atrial mass measuring 5.5x4.5x3.7 cm in aggregate was surgically removed. The histopathologic evaluation of the left atrial-ventricular mass showed low cellularity and abundant extracellular matrix, papillary projections from the surface, and spindle-shaped and stellate cells without mitotic figures surrounded by a myxoid stroma (Figures 4-6). Alcian blue 2.5 pH staining showed that the extracellular matrix contained abundant acid mucin and mucopolysaccharides (Figure 7). Calretinin staining and immunohistochemistry were positive for calretinin and cluster of differentiation 34 (CD34) and were negative for cluster of differentiation 68 (CD68) (Figure 8). Given the clinical and histopathologic findings, the patient was diagnosed with an atrial myxoma.

**Figure 9:**
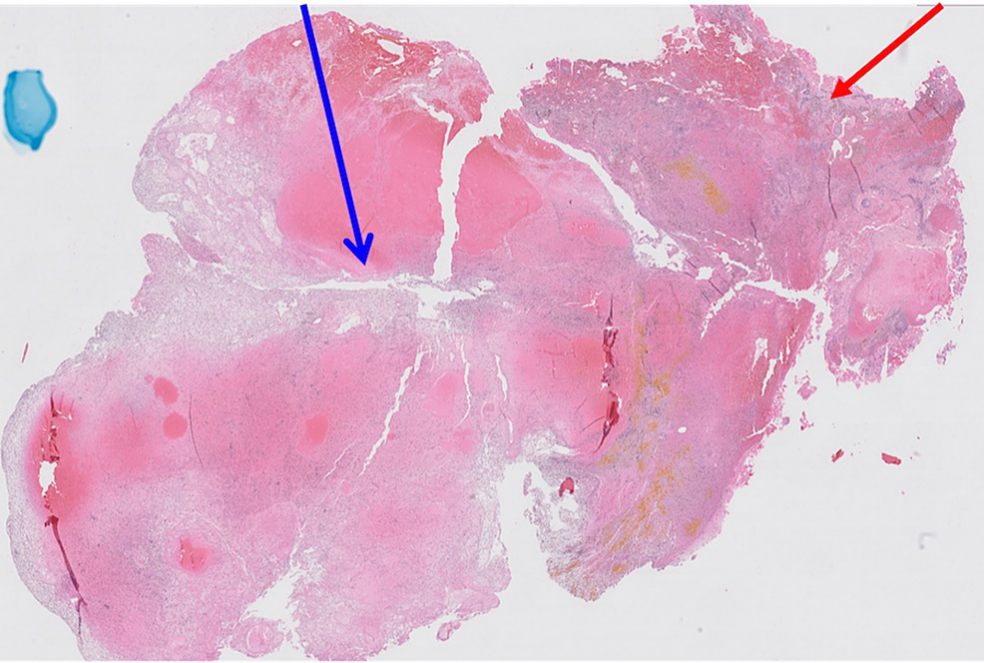
A low-magnification view of the left occipital lobe lesion shows myxoid regions (blue arrow) and areas of acute, subacute, and chronic hemorrhages (red arrow).

**Figure 10:**
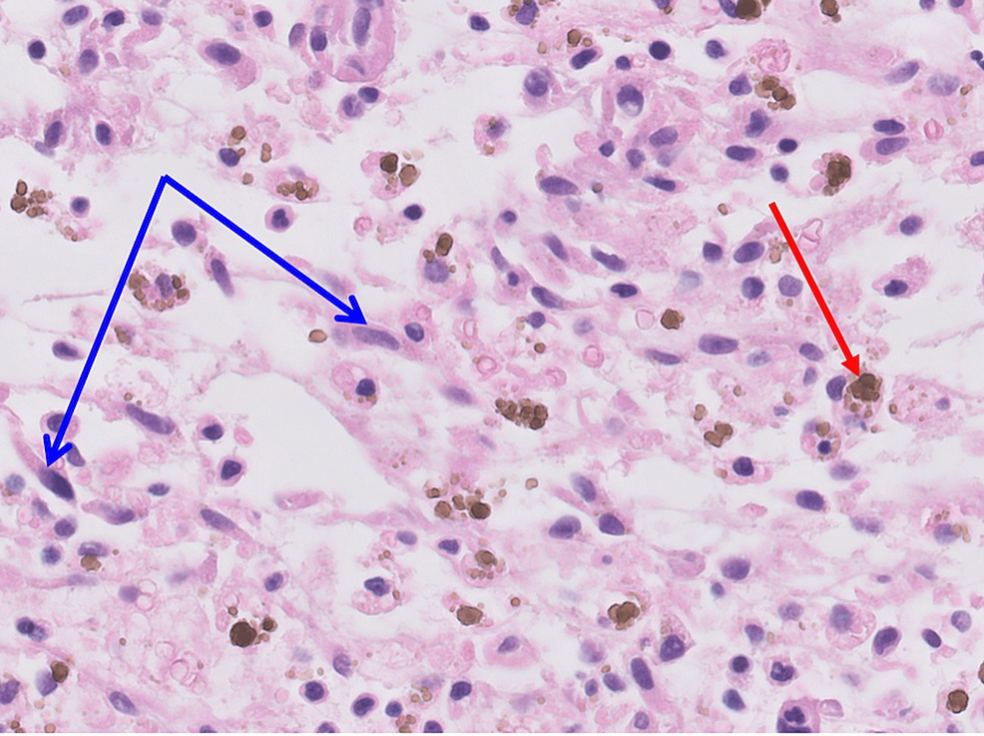
A high-magnification view of the left occipital lobe lesion shows spindle-shaped and stellate cells (blue arrows) and macrophages containing hemosiderin (red arrow).

**Figure 11:**
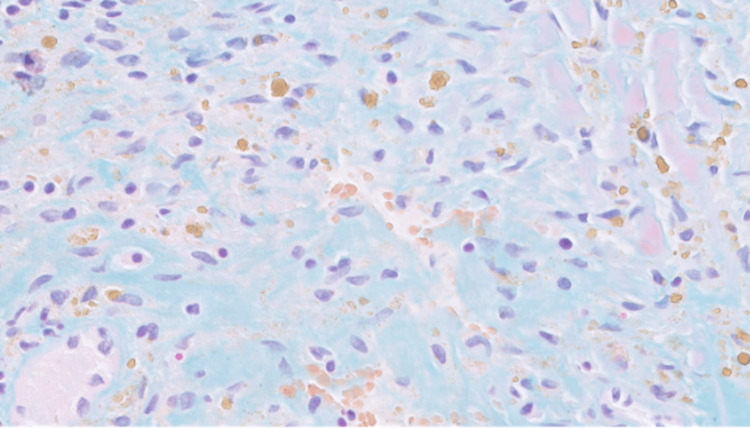
Alcian blue pH 2.5 stain of the left occipital lobe lesion. The extracellular matrix contains abundant acid mucin and mucopolysaccharides.

**Figure 12:**
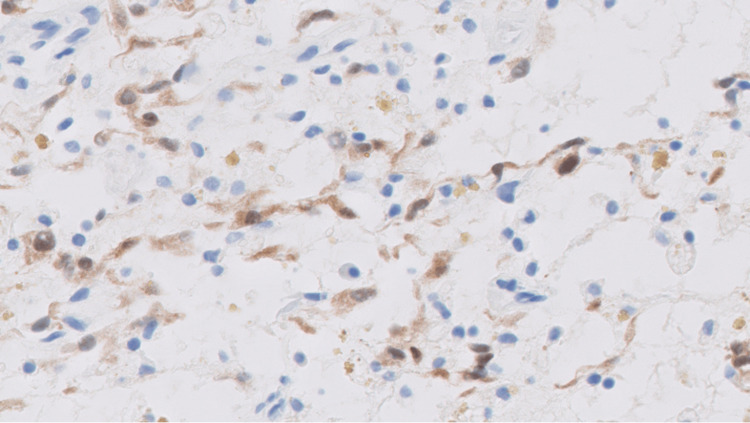
Calretinin stain of the left occipital lobe lesion. Immunohistochemistry is positive for calretinin, CD34, and macrophages and negative for CD68. CD34: cluster of differentiation 34; CD68: cluster of differentiation 68

The left occipital lobe lesion was resected 2.5 months later. The histopathologic evaluation of the lesions showed myxoid regions, areas of acute, subacute, and chronic hemorrhages, spindle-shaped and stellate cells, and macrophages containing hemosiderin (Figures 9-10). Alcian blue 2.5 pH staining showed that the extracellular matrix contains abundant acid mucin and mucopolysaccharides (Figure 11). Calretinin staining and immunohistochemistry were positive for calretinin, CD34, and macrophages and were negative for CD68 (Figure 12).

An embolic atrial myxoma was the most likely cause of the patient’s brain lesions. The histopathologic comparison between the left occipital lobe lesion and left atrial-ventricular mass suggested they were of the same origin. The similarities between them included the presence of spindle-shaped and stellate cells, myxoid regions, Alcian blue pH 2.5 positivity (more positively stained in the atrial mass), calretinin positivity, CD34 positivity, and CD68 negativity. The exact nature of the multiple, smaller lesions in his brain were not evaluated, and they could have been the result of either thromboemboli or myxoma emboli.

## Discussion

Atrial myxomas are the most common primary cardiac neoplasm in adults, consisting of 30-50% of all benign cardiac tumors [6]. They are of mesenchymal cell origin and benign and have a median age of incidence of 49, but they may present in teens and young adults [6,7]. Myxomas can occur in the ventricles, but approximately 75% arise in the left atrium and are usually solitary [6]. If a myxoma occurs on the left atrium, it may obstruct the mitral valve, leading to mitral valve dysfunction, syncope, or sudden death [8]. Although they do not metastasize, embolization is possible, as was true in the present case [9].

Like the findings in Figures 4-6, atrial myxoma histology often shows spindle-shaped nuclei with a minimal cytoplasm and extracellular material containing myxoid glycosaminoglycans and other materials [10]. Figure 5 also shows evidence of the friable, papillary nature of the mass, which is suggestive of its potential to embolize. Alcian blue staining in Figure 7 confirmed that the extracellular matrix contained abundant myxomatous materials made up of mucosubstances and acetic mucins. Calretinin, a calcium-binding protein, can be important in diagnosing myxomas as it is positive in approximately 73.9% of the cases (Figure 8) [11]. CD34 positivity is generally present in most cells; however, it is a sensitive marker for assessing myxomas [12]. In addition, myxomas are commonly immunoreactive for vimentin, S100, nonspecific enolase, factor VIII, and CD31 [13]. CD68 positivity is commonly used to identify cells with a macrophage lineage [14]. As many neoplasms with metastatic potential overexpress macrophage antigens, CD68 is an important marker for identifying cells in a prometastatic state [14]. In this case, its negativity was reassuring that the sampled tissue was benign [14].

The largest data series on atrial myxomas to date by Lee et al. evaluated 74 cases of atrial myxomas; only nine (12%) of these total cases presented with neurologic manifestations [15]. Other data series summarized have suggested that neurological symptoms may be present in 26% to 45% of cases [16-20]. Of the nine cases described by Lee et al., eight presented with ischemic central nervous system (CNS) infarcts, one presented with CNS hemorrhages, one presented with internal cerebral artery aneurysmal dilation (due to myxomatous proliferation inside the vascular lumen), and two presented with other systemic myxomatous emboli [15]. None of the nine patients demonstrated symptoms of mitral valve obstruction or constitutional effects, which suggests that patients with atrial myxomas who develop neurological complications may lack concomitant cardiac symptoms [15].

The mean Modified Rankin Score (mRS), which assesses stroke victim disability on a scale of zero (no disability) to five (disability requiring care for all needs), on hospital discharge was two, with a range of two to four, for all nine patients who presented with neurologic manifestations in the study by Lee et al. [15]. The follow-up mean mRS was one with a range of zero to three for six of the nine participating patients [15]. These data suggest that the long-term outcomes for patients suffering strokes secondary to myxomatous emboli are relatively favorable.

Patients in the same study by Lee et al. who presented with myxomatous emboli to the CNS were between 17 and 70 years old, with a mean age of 48.5 years [15]. There was also a two-to-one female-to-male ratio in these cases [15]. The mean diameter of the atrial myxomas evaluated was 2.7 cm - ranging from 0.4 to 6.5 cm. These data contribute to the understanding that the mobility of a myxoma, not size, is associated with embolic potential [15].

## Conclusions

Atrial myxomas are the most common primary neoplasm of the heart. In addition to effects on the heart and mitral valve, adverse effects of atrial myxomas on the brain include embolization of either fragments of the neoplasm or thrombus formed in the atrium in the setting of stasis due to obstruction by the lesion. Most emboli to the CNS are due to thromboembolism, atheroembolism, or vegetation embolism. However, when considering an embolic stroke, atrial myxomas should be considered in the differential diagnosis in the setting of concomitant cardiac abnormalities. Generally, multiple hemorrhagic or ischemic infarctions are due to neoplastic (e.g., metastasis) or non-neoplastic (e.g., traumatic or vascular) causes. Most brain neoplasms are metastatic in origin, and the neoplasms that most frequently metastasize to the brain are from the lungs. A cardiac mass seen on imaging is likely a thrombus or vegetation but may be a cardiac neoplasm. Histology is important to recognize a myxoma, which typically shows spindle-shaped nuclei with minimal cytoplasm and extracellular material containing myxoid glycosaminoglycans and other materials. Alcian blue staining, calretinin staining, and immunohistochemistry play essential roles in diagnosis. This case was remarkable due to the patient’s late presentation, the large size of the atrial myxoma, the presence of abundant cerebral hemisphere and cerebellar lesions, and the histologic comparison of the heart and brain lesions, including the identification of the embolic myxoma within the brain blood vessels and its damage to brain blood vessels.
